# Fluid–Structure Interaction for Biomimetic Design of an Innovative Lightweight Turboexpander

**DOI:** 10.3390/biomimetics4010027

**Published:** 2019-03-22

**Authors:** Ibrahim Gad-el-Hak

**Affiliations:** Department of Mechanical Engineering, École Polytechnique de Montréal, Montréal, H3C 3A7, Canada; gadelhakibrahim@gmail.com

**Keywords:** biomimetic composite blade, nature-inspired, organic Rankine cycles, computational fluid dynamics, finite element analysis, fluid–structure interaction, bird feather, fiber orientation, turboexpanders

## Abstract

Inspired by bird feather structures that enable the resistance of powerful aerodynamic forces in addition to their lower weight to provide stable flight, a biomimetic composite turbine blade was proposed for a low-temperature organic Rankine cycle (ORC) turboexpander that is capable of producing lower weight expanders than that of stainless steel expanders, in addition to reduce its manufacturing cost, and hence it may contribute in spreading ORC across nonconventional power systems. For that purpose, the fluid–structure interaction (FSI) was numerically investigated for a composite turbine blade with bird-inspired fiber orientations. The aerodynamic forces were evaluated by computational fluid dynamics (CFD) using the commercial package ANSYS-CFX (version 16.0) and then these aerodynamic forces were transferred to the solid model of the proposed blade. The structural integrity of the bird-mimetic composite blade was investigated by performing finite element analysis (FEA) of composite materials with different fiber orientations using ANSYS Composite PrepPost (ACP). Furthermore, the obtained mechanical performance of the composite turbine blades was compared with that of the stainless steel turbine blades. The obtained results indicated that fiber orientation has a greater effect on the deformation of the rotor blades and the minimum value can be achieved by the same barb angle inspired from the flight feather. In addition to a significant effect in the weight reduction of 80% was obtained by using composite rotor blades instead of stainless steel rotor blades.

## 1. Introduction

Growing populations and increasing industrialization have globally led to an exponential increase in energy demands. In the meanwhile, growing concerns over global warming and the environmental issues of conventional energy resources, such as oil, gas, and coal, are initiating the need to meet global energy demand from renewable energy resources and to improve energy efficiency by recovering low-grade heat sources. The organic Rankine cycle (ORC) has been recently used as a low-grade heat recovery system. It is a technology that operates correspondingly to the steam Rankine cycle, except that steam is replaced with organic working fluids. This organic working fluid has a lower boiling point compared to that of steam, and is consequently able to use waste heat sources to drive a turbine for power generation [1]. The main two parameters which determine the performance of an ORC system are working fluid selection and expander design. A wide range of research conducted in the area of working fluid selection was basically performed to calculate the cycle efficiency and output power for different working fluids through thermodynamic analysis for an ORC cycle [2,3,4,5,6]. Moreover, Darvish et al. [7] tested nine working fluids, namely, R134a, R123, R227ea, R245fa, R600, R600a, iso-pentane, *n*-pentane, and toluene to identify the best performing fluid for low-temperature ORC applications. They performed the thermodynamic model for ORC system which has a source temperature of 120 °C to evaluate thermodynamic parameters such as, output power and exergy efficiency, within system constrains. They found that refrigerant R600a produced highest exergy efficiency of 20.3% among other working fluids. In addition, R600a has a low global warming potential (GWP) of three and zero ozone depleting potential (ODP) and thereby its environmental impact is lower. In addition to an expander design was selected based on its efficiency, targeted power output and size which were determined through the preliminary design process of an expander [8,9,10,11]. Radial turboexpanders are preferred for ORC systems due to their high tip speed, which contributes positively in stage-specific work [6]. The rotational turbine speed is nevertheless limited by the strength of materials at the wheel periphery due to the centrifugal forces. Moreover, the rotational speed should be selected to obtain the blade speed ratio (*U_2_/C_0_*) near to 0.7 for better isentropic efficiency. However, technical analysis is an essential first step that should be considered in the design process of a cycle, but the economic perspective of a cycle is crucial to estimate the energy price. Generally, the feasibility of implementing energy-efficient technology is found to be sensitive to the cost of equipment more than to the expected energy costs [1]. The expander is the most expensive component in an ORC system as it costs around 50% of total investment costs [1].

Composite materials can be used to produce innovative low-cost expanders for ORC systems. There are benefits to manufacturing composite turboexpanders for an ORC system due to (i) their lower weight than that of stainless steel turboexpanders lead to reduce centrifugal loads acting on the turbine rotor due to its high rotational speed, (ii) their corrosion-resistant properties provide an advantage for operation with a two-phase flow without the occurrence of corrosion in the turbine blades, (iii) their low thermal conductivity will help to reduce the heat loss from the turbine by maximizing the use of the flow energy, and (iv) they have a design flexibility which can be used easily for the twisted turbine blade. Moreover, the fiber orientations of unidirectional fibrous composites play a significant role in increasing its stiffness and strength by aligning the fibers parallel to the direction of loading. However, choosing of a fiber angle is a challenging task when the load is applied into two different directions, such as bending and torsional loads.

### Biomimetic Design of Turboexpanders

**Figure 1:**
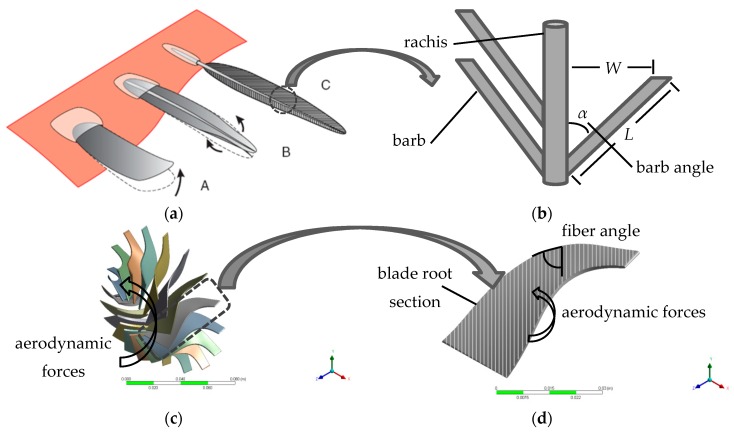
(**a**) Three stages in the development of flight feathers from simple scales in birds [12]: (A) a simple scale tends to bend upwards in response to air pressure, (B) a longitudinal stiffening ridge resists upward bending, and (C) stiffening ridges in the vanes resist the curling tendency. (**b**) Schematic diagram of the mature feather branch morphology: vane width (W), barb length (L), and barb angle with respect to the rachis (α). (**c**) Geometry of the rotor blades for a radial inflow turbine and (**d**) schematic diagram of the proposed fiber orientation inspired by bird feathers.

Biomimicry of flight wings of birds can be incorporated to design the composite turbine blade of a turboexpander, with fiber orientations mimicking the bird’s feathers structure. A bird’s flight feather has elaborate structures that could only have evolved to meet the need to resist bending and torsion in a wing, and to transmit aerodynamic forces to the shaft (rachis) [12]. During the developmental stages of the bird wing, the flight feathers grow up to be structurally able to resist the aerodynamic forces as shown in Figure 1a. Between developmental stages (B) and (C), a stiffening shaft (rachis) along the axis of the feather resists both bending and torsion, but does not prevent the vanes from twisting up at the edges in response of air flow, which become stiffer at both edges as the twisted angle of the feather vane is reduced by configuration of an array of parallel ridges (barbs) as indicated in Figure 1b. Thereby, the fluid–structure interaction between the flowing air and the feather is reduced. By analogy, this is the same problem in a composite turbine blade. Due to the elasticity of a composite turbine blade and the aerodynamic forces around the blade, the interaction between them leads to twisting of the blade tip, and the blade angle changes from the optimum design point (Figure 1c), thus resulting in a deteriorated performance of the turboexpander. Here, we use the orientation of feather barbs as inspiration to design a composite turbine blade for ORC turboexpanders as bird feathers have several features that make them ideally suited for flight (Figure 1d). The direction of the barbs from the leading and trailing edge increases the torsional stiffness of the feather and reduces the twisted angle during the interaction with the flowing air. Thus, using unidirectional fibrous composites with an acute fiber angle, as indicated in Figure 1d, in manufacturing the rotor turbine blades would have a significant contribution to reducing its deformed twisted angle as well as reducing its weight.

**Figure 2:**
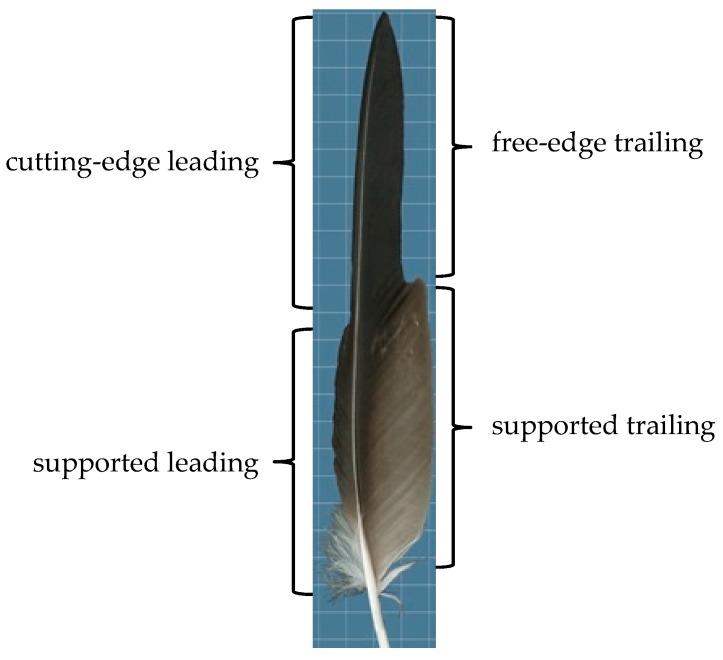
Four vane partitions recognized by aerodynamic functions: cutting-edge leading vanes, supported leading vanes, free-edge trailing vanes and supported trailing vanes.

Understanding the flight feather vanes structure can be helpful to create lightweight as well as structurally robust turboexpanders because the barb angle is adjusted to meet the variations of aerodynamic forces along the flight vane. Thereby, the flight feather vanes can be classified into four main categories based on their aerodynamic functions as shown in Figure 2 [13]: (i) cutting-edge leading vanes function as the cutting edge of an aerofoil during flight; (ii) free-edge trailing vanes function as the unsupported trailing edge of an aerofoil during flight; (iii) supported leading vanes; and (iv) supported trailing vanes overlap with each other to create a continuous surface of the wing. The geometry of feather barbs (barb length and barb angle) defines feather vane asymmetry and vane rigidity, which are both critical for a feather’s aerodynamic performance. For instance, two feathers with the same vane width could vary extensively in barb geometry which is reflected on their response to aerodynamic forces. Thus, Feo et al. [13] analyzed feather barb geometry of 60 species of modern flying birds including the four flight feather vanes. They measured barb angle and barb length for each feather of leading and trailing vanes at 25% and 50% of total vane length from the tip of the feather. Among the analyzed feathers, they found that the barb angles were varied based on the function of flight feathers. They observed that both barb angle and barb length significantly differed between cutting-edge and supported leading vane partitions, but not between trailing vane partitions. They found that free-edge trailing vanes, supported trailing vanes and supported leading vanes coincided with each other in morphospace due to their similar ranges of large barb angles (30–50°). By contrast, cutting-edge leading vanes were classified to a distinct region of morphospace characterized by small barb angles (less than 24°). They concluded that small barb angles can contribute to vane rigidity (i.e., resistance to aerodynamic forces during flight) in contrast to larger barb angles help increase the flexibility of a vane [14].

Differences in the mechanical behavior of feathers are strongly correlated to differences in their morphology and function. Moreover, Enno et al. [14] examined the functional morphology of feather vanes by combining morphological examination with mechanical tests. Their practical work was carried out on feathers from both wings of the pigeon *Columba livia*. They measured both the length of the vane and the branching angles of barbs from leading and trailing edge at three points beginning from the base to the tip, located at one-quarter (base), half (middle), and three-quarters (tip). They found that the mean branching angles for the leading edge at base, middle and tip were 37 ± 10°, 28.5 ± 6°, and 23 ± 7.5°, respectively. While the mean branching angles were relatively larger in the trailing edge, these values at base, middle and tip became 48.6 ± 10°, 40.4 ± 4°, and 35 ± 4°, respectively. In addition, the mechanical tests were performed on each trailing edge vane of the selected feathers at three different locations—the base, middle, and tip—to examine their mechanically resistance. They applied the loads on the trailing vane as mimicking aerodynamic forces until the failure of vane occurred. They concluded that the tip region of each vane resisted greater moments than both the middle and base due to the lower branching angles of the barbs, which helped to produce the stiffer structure of the feathers. 

It is obvious that the vanes of the bird flight feathers have mechanically competent grid texture whose behavior is controlled by their geometry (barb angle), giving a structure which combines lightness, flexibility, and mechanical competence. 

Here, the structure of flight feathers is utilized to propose lightness and robust and cheaper turboexpanders for a low-temperature ORC system. The biomimetic composite turbine blade was molded as an unidirectional fibrous composite with different fiber orientations (20°, 30°, 40°, 50°, and 60°) by using ANSYS Composite PrepPost (ACP) to select the optimum fiber angle. Consequently, the fluid–structure interaction (FSI) for the biomimetic composite turbine blade was investigated numerically by performing integrated computational models of the proposed blade to evaluate the structural integrity in terms of the tip deflection and von Mises stress due to centrifugal, thermal, and aerodynamic loads. The FSI model was obtained by coupling a finite element analysis (FEA) based on computational solid dynamics (CSD) andcomputational fluid dynamics (CFD). The CFD simulations based on a three-dimensional (3D) Reynolds-averaged Navier–Stokes (RANS) model were performed for the turboexpander to obtain the thermal and aerodynamic loads through the blade surface.

## 2. Numerical Simulations

In order to investigate the FSI of the biomimetic composite blade, a computational fluid domain for the turbine was performed to obtain the aerodynamic and thermal loads and thereby these loads can be inserted to a structural model of the proposed blade. Three-dimensional CFD simulations for a turboexpander were performed by using a commercial package ANSYS-CFX (version 16.0, ANSYS, Inc., Cannonsburg, PA, USA) as well as static structural analysis were performed for the proposed composite blade using a commercial package ANSYS Static Structural and ANSYS Composite Pre/Post (ACP) (version 16.0, ANSYS, Inc.). 

### 2.1. CFD Simulations

In radial turboexpanders, the flow is mainly 3D due to the complexity of the turbine blade geometry. Hence, turbine 3D CFD simulation is required to determine aerodynamic forces and temperature distribution through the turbine blade profile. Using a 3D CFD model, three governing equations of fluid dynamics—the continuity, momentum in three-directions (*x*, *y*, and *z*), and energy equations—were numerically solved by using ANSYS-CFX (version 16.0). The detailed geometry of turbine blade was generated through ANSYS Blade-Gen (version 16.0, ANSYS, Inc.) for both the stator and rotor. After that, the solid model of the turbine blade was generated; the fluid domain was discretized using ANSYS Turbo-Grid (version 16.0, ANSYS, Inc.) into structured (hexahedron) meshes. Three-dimensional RANS steady compressible flow simulations with standard *k*–ω turbulence model was solved in ANSYS-CFX for a single stage radial inflow turbine, which is normally used in the Sundstrand Power Systems T-100 Multipurpose Small Power Unit [15]. The obtained numerical results from the turbine model with air as working fluid were compared with the experimental data [15] in order to validate the CFD turboexpander model. Subsequently, the turboexpander model was implemented in low-temperature ORC system with scaling boundary conditions from air to R600a working fluid. For this ORC system, the inlet and outlet turbine temperatures were set to 370 and 313 K, respectively; and the turbine rotational speed was selected to maintain the optimum blade speed ratio of 0.7. The obtained thermal and aerodynamic loads from the turbine model with R600a were used to investigate the aeroelasticity of the proposed composite turbine.

#### 2.1.1. Geometry

**Figure 3:**
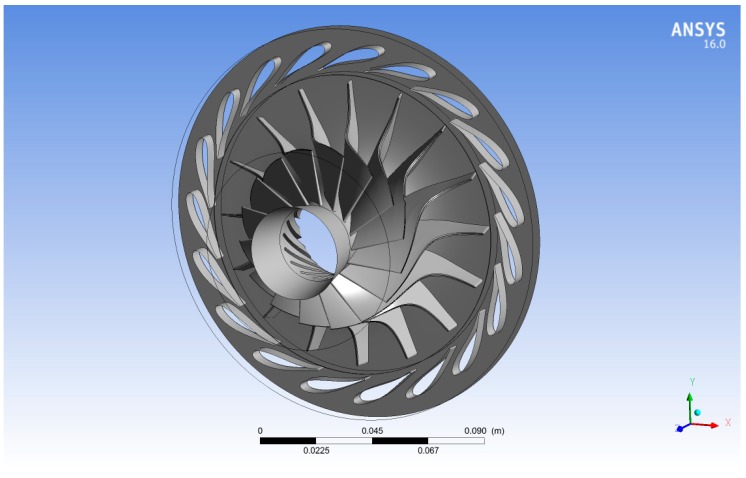
Three-dimensioanl model of the radial inflow turbine.

The 3D model was generated in ANSYS Blade-Gen (version 16.0) based upon published geometrical information [16]. The rotor and stator blade profiles, including the wrap angle, blade angle, and blade thickness through its meridional axis, were used to define a solid model for the rotor and the stator blades. Figure 3 shows the final 3D model of the radial inflow turbine which consists of 16 rotor blades and 19 stator blades.

#### 2.1.2. Mesh for Fluid Analysis

**Figure 4:**
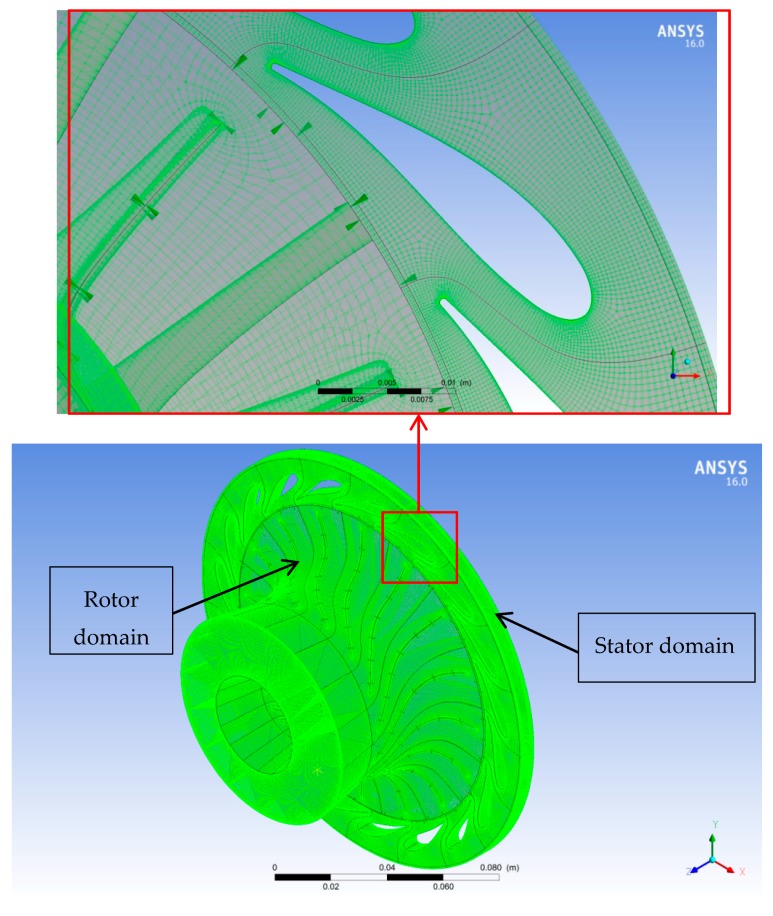
Computational meshes of the rotor and stator domains.

The structured (hexahedron) meshes for both the rotor and stator flow passages were generated using ANSYS Turbo-Grid (version 16.0). Automatic topology and meshing (ATM-optimized) was adjusted for the rotor and nozzle domains to maintain high mesh quality as blade shape changes from hub to shroud. The element size refinement was controlled in the boundary layer by selecting the method proportional to mesh size with a setting factor base and factor ratio of 0 and 1.6 respectively, to ensure the meshes able to capture the turbulent flow structures and the boundary layer effects near the walls. A turbulence model chosen was standard k–ω to capture the turbulence phenomena. The nondimensional grid spacing (*y*^+^) at the wall is required to be near 1.0 for the k–ω standard turbulence model; however, the typical value of *y*^+^ at the rotor blade was 9.8 in case of air. Subsequently, the option of automatic wall functions was chosen, which allows the k–ω model to obtain good predictions albeit the value of *y*^+^ is larger than 1.0. The total number of elements for the rotor and nozzle mesh was 3,272,048 and 4,272,122 elements, respectively. The 10 layers of elements were inserted in the shroud tip clearance of 0.23 mm in radial direction to 0.4 mm in axial direction. The computational meshes for the rotor and stator domains used for the validation of the CFD model are shown in Figure 4.

#### 2.1.3. CFD Model Setup

**Figure 5:**
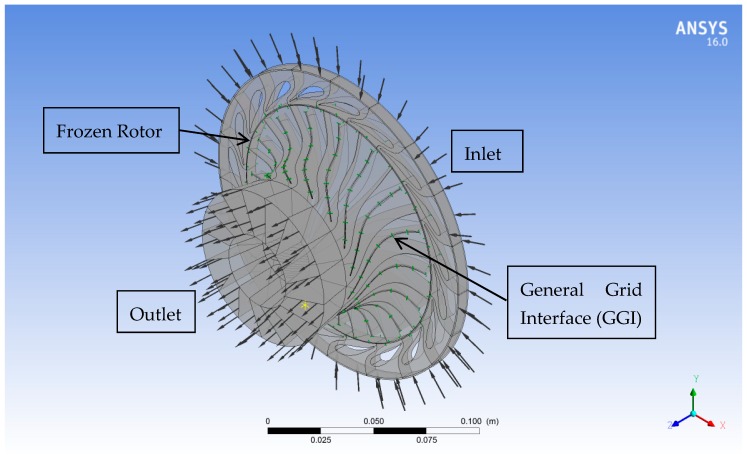
Boundary conditions for the full turbine model including the entire rotor and stator blades.

Boundary conditions, interfaces, properties of working fluid, and a turbulence model are required to define each computational domain to run the simulations. In this study, a full turbine model including the entire rotor and the stator blades was selected to carry out the flow simulations. In order to model the interaction between the rotating (rotor) and stationary (stator) domains, the frozen rotor method was defined at the interface between them. Moreover, general grid interface (GGI) was defined at the shroud tip to connect nonconfirming grids. In addition, boundary conditions were required to define at the borders of each domain to solve the computational domain. Inlet boundary conditions for the turbine model were the total pressure and the total temperature whereas the static pressure was defined at the turbine outlet. Two sets of boundary conditions were used in this study as shown in Table 1. The CFD flow simulation was performed with the first set in order to validate the turbine model with the experiments that conducted by Jones [15] using air working fluid. In addition, the CFD turboexpander model was run with boundary conditions of R600a as a result of implementing it in ORC system to calculate the aerodynamic forces. This is due to the fact that organic fluids should be considered as a real gas to predict accurate simulations; Peng–Robinson equation of state [17] is implemented in the solver ANSYS-CFX to determine the real gas properties of R600a. All wall boundaries were imposed a no-slip condition, smooth wall, and adiabatic. The boundary conditions of the full turbine model are shown in Figure 5. Steady-state simulations were run until root mean square (RMS) residuals were achieved to below 10^−6^.

### 2.2. FEA Simulations

After the aerodynamic forces calculated along the blade surface, 3D turbine blade FEA simulations were performed to evaluate the mechanical performance parameters of the biomimetic composite turbine blade during turbine operations. In this analysis, von Mises stresses and blade deformation were obtained by ANSYS Static Structural and ANSYS Composite Pre/Post (ACP) to ensure that the biomimetic turbine blades are structurally robust.

#### 2.2.1. Mesh for Structural Analysis

In order to compare the mechanical performance between the turboexpander manufactured in stainless steel and with that of composite rotor blades, two cases have been studied. In the first case, the whole turbine was modeled as 3D solid model of stainless steel; thereby its geometry was transferred to ANSYS Static Structural to create its mesh and define its material’s properties. 

**Figure 6:**
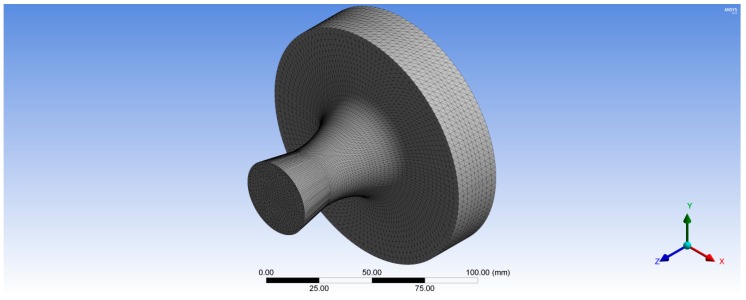
Three-dimensional structural mesh for the wheel turbine hub with 175,487 nodes.

**Figure 7:**
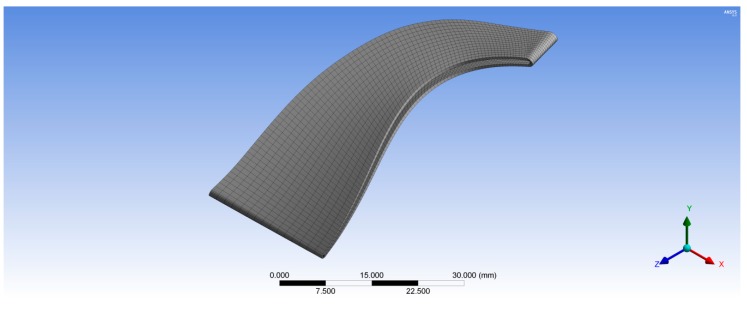
Three-dimensional structural mesh for one rotor blade.

**Figure 8:**
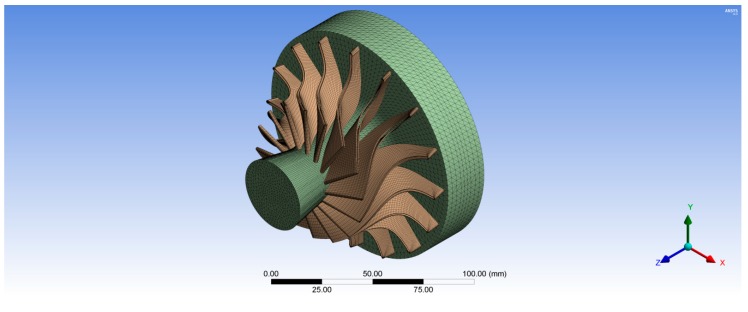
Three-dimensional structural model for the turbine including the composite rotor blades and the stainless steel wheel hub.

Figure 6 shows the unstructured tetrahedral mesh for the wheel hub with 175,487 total nodes. Whereas, the structured hexahedral mesh was created for the rotor blades with 38,248 total nodes for one rotor blade as shown in Figure 7. In the second case, the rotor turbine blades were modeled as a composite material with different fiber orientations while, the wheel turbine hub was modeled as stainless steel. Hence, the rotor turbine blades generated in BladeGen were transferred to ACP (Pre) to create the mesh for their blade thickness as shown in Figure 7; whereas the 3D model of the wheel hub was transferred to ANSYS Mechanical Model (version 16.0, ANSYS, Inc.) to create its tetrahedron mesh with 175,487 total nodes as shown in Figure 6. The composite rotor blades were assembled with its hub in ANSYS Static Structural as shown in Figure 8. 

#### 2.2.2. Turbine Composite Materials 

**Figure 9:**
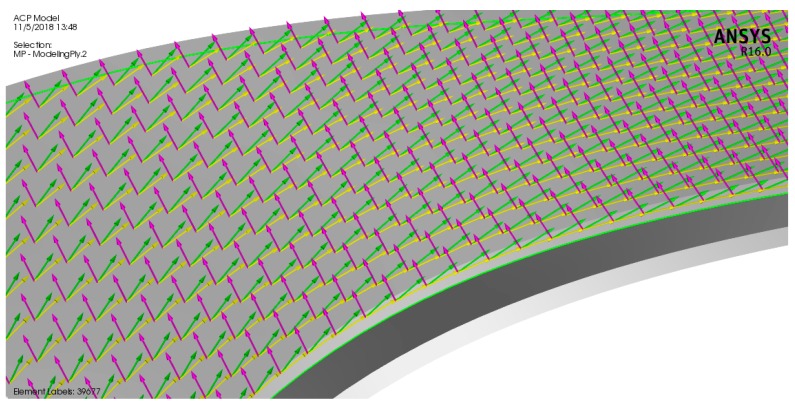
An ANSYS Composite PrepPost (ACP) composite model for the rotor blade with fiber orientations of 20° from the blade edge.

**Figure 10:**
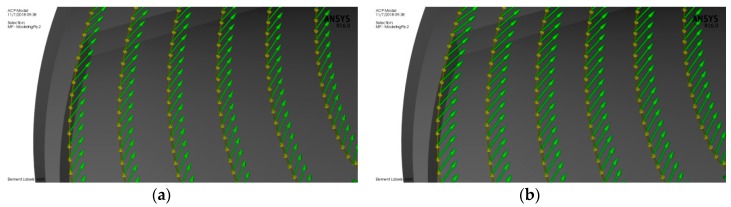

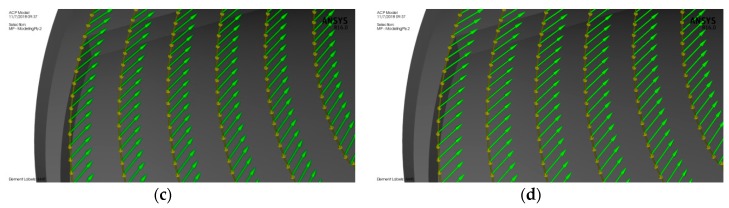
An ACP composite model for the rotor blade with different fiber orientations:(**a**) 30° from the blade edge, (**b**) 40° from the blade edge, (**c**) 50° from the blade edge, and (**d**) 60° from the blade edge.

ANSYS Composite Pre/Post (ACP) was used to assign the composite materials to the rotor blades. Epoxy carbon UD (unidirectional) prepreg was selected for the composite rotor blades because its glass transition temperature around 120 °C, which is below the inlet turbine temperature of 97 °C. Table 2 shows the properties of the materials used in modeling the turbine. An ACP composite model of the bird-mimetic composite blade is shown in Figure 9. The reference direction for the composite material was considered parallel to the blade edge which is indicated by yellow arrows, the fiber direction from the reference is indicated by green arrows, and the composite layup direction is indicated by pink arrows. The turbine consists of 16 rotor blades and each blade has been modeled with 0.5 mm of turbine material on each side of the blade, pressure side and suction side. Figure 10 shows the composite material model of the blade with different fiber orientations 30°, 40°, 50°, and 60° from the blade root section.

#### 2.2.3. Fluid–Structure Interaction Model Setup

**Figure 11:**
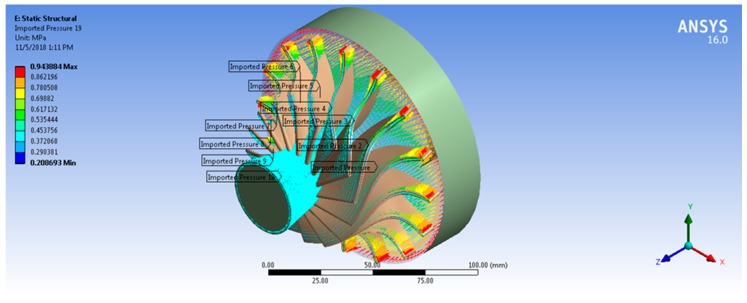
Imported pressure on the rotor blades and the wheel hub.

**Figure 12:**
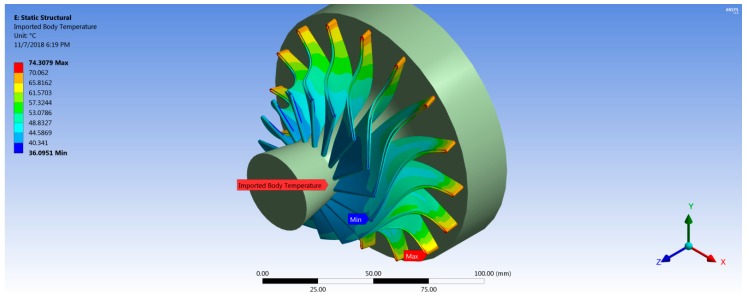
Imported temperature distribution for the rotor blades.

**Figure 13:**
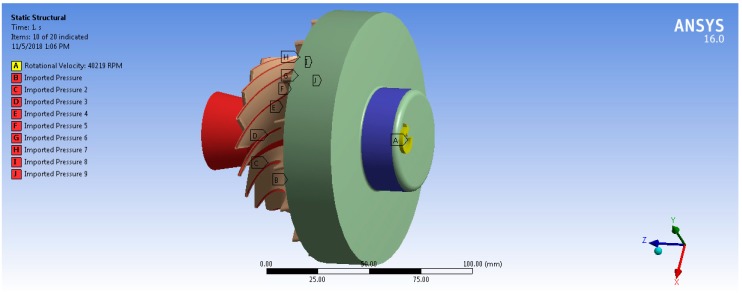
Rotational velocity (A), cylindrical support (blue surface) for the turbine model and imported pressure on the rotor blades (B, C, D, E, F, G, H, I, J).

Fluid–structure interaction can be considered as the result of the mutual interaction of three main disciplines: dynamics, solid mechanics, and aerodynamics [18]. The one-way FSI system was used in this analysis. For the static structural analysis, the boundary conditions and loads are defined for the model in order to determine the stresses and deflections in the turbine. These loads are caused by the temperature and pressure of the working fluid, and turbine rotational speed. Based on Ansys-CFX simulation results, the aerodynamic pressure force on the rotor blades and thermal loads due to working fluid temperature are transferred to a static structural model and solved for equivalent von Mises stress and total deformation. Therefore, ANSYS is mapping the forces on each node from CFX into the mechanical node. Consequently, the position of the blade geometry in CFX module and Static Structural module must be same with reference to the global coordinate system. The results showed that 97% of mechanical nodes were mapped to the CFD surface. Both Figure 11 and Figure 12 show the imported pressure and temperature distributions on the rotor and the hub wheel from CFX into the static structural model. In order to model the centrifugal stresses, the rotational speed and the cylindrical support were applied on the wheel hub as shown in Figure 13. The rotational turbine speed was 40,219 rpm as indicated in Table 1. The cylindrical support limits the motion of the turbine wheel in the radial and axial directions and it is free in the tangential direction. 

## 3. Results and Discussion

### 3.1. Validation of the CFD Model

The comparison of the mass flow and the total to static efficiency ($\eta_{t-s})$ as defined by Equation (1) between the experimental data that conducted on the turbine by Jones [15] and CFD results are summarized in Table 3. It can be concluded that total-to-static efficiency matches very well (1.4%) near the design point (pressure ratio (Pr) = 5.73 and $\eta_{t-s}$ = 86.4%). In addition, the mass flow rate obtained from the experiment and CFD match well (2.7%) at the design point ($\dot{m}$ = 0.33 kg/s), indicating that the CFD model is sufficient to predict the turbine performance near the design point. Consequently, the CFD model was run with boundary conditions of R600a to determine the aerodynamic and thermal loads that required in the structural analysis of the turbine. The obtained numerical total-to-static efficiency in case of R600a is 86.2% which indicates that the selected rotational speed coincides with the optimum value of speed ratio:

(1) $$\eta_{t-s}=\frac{h_{01}-h_{03}}{h_{01}-h_{3_{ss}}}$$

where $h_{01}$ is the total enthalpy at the inlet of the turbine, $h_{03}$ is the total enthalpy at the turbine outlet, and $h_{3ss}$ is the static isentropic enthalpy at the turbine outlet. 

**Figure 14:**
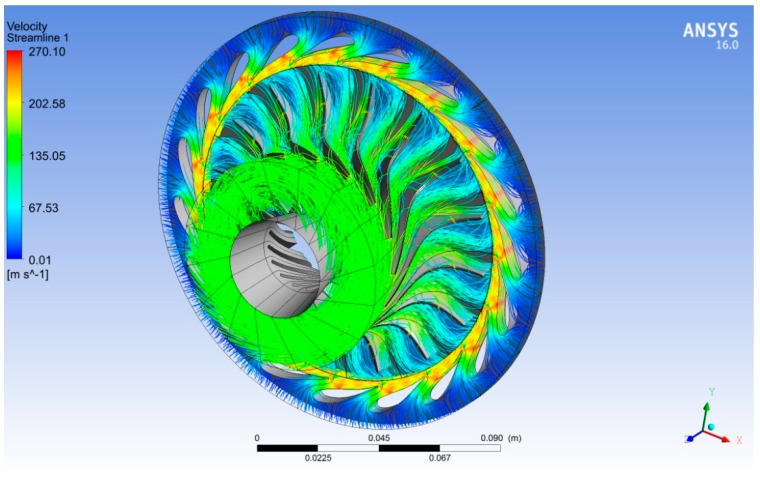
Three-dimensional velocity streamlines in case of R600a as a working fluid at 40,219 rpm.

**Figure 15:**
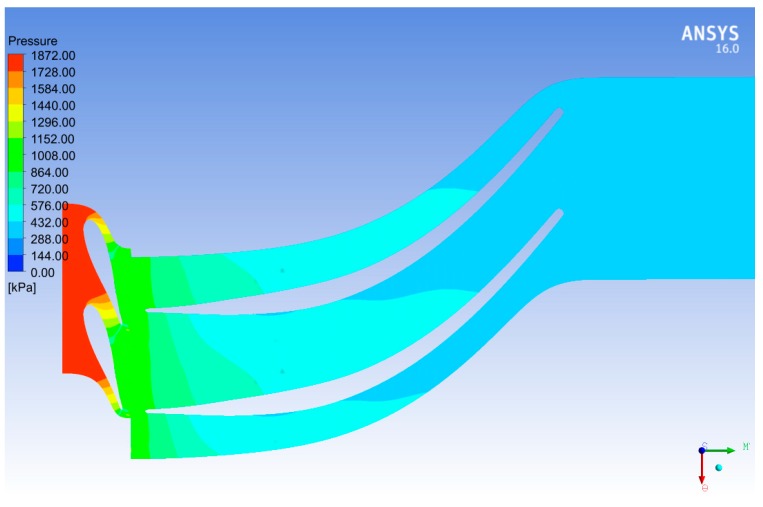
Two-dimansional static pressure contours through the stator and rotor blades at 50% span.

Figure 14 shows 3D streamlines of the velocity around the rotor and stator blades in case of R600a as a working fluid. We observed that the maximum velocity of R600a occurred at the nozzle outlet due to the acceleration effect of the nozzle throat. In addition, the flow velocity was relatively high at the rotor outlet due to the rotation of the rotor. When the energy of the fluid was extracted by rotating of the rotor blades, then the flow left the rotor outlet with less pressure as shown in Figure 15. 

**Figure 16:**
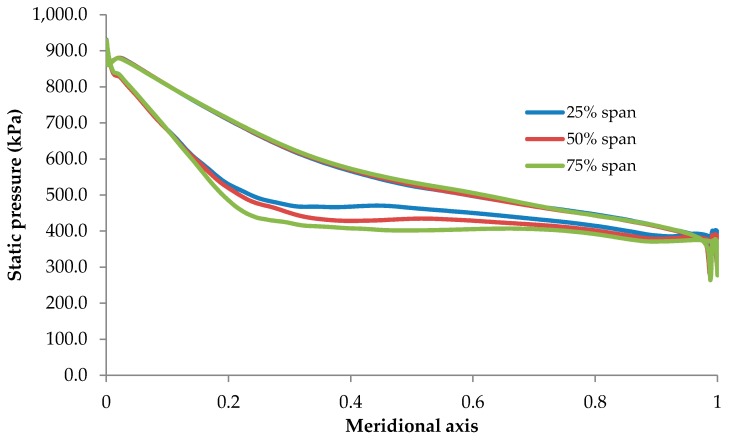
Pressure distribution along the rotor blade for R600a at three different locations: 25% span, 50% span, and 75% span.

In order to import the aerodynamic forces from the CFX into the mechanical module, it is required to obtain the effect of R600a forces on the rotor blades. Figure 16 shows the samples of pressure distribution at three locations of the rotor blade heights 25%, 50%, and 75% span. The rotor blade appeared to encounter high loading at its leading edge accompanied with declining loading toward the trailing edge which may cause structural issues for the blade integrity.

### 3.2. Static Structural Results

**Figure 17:**
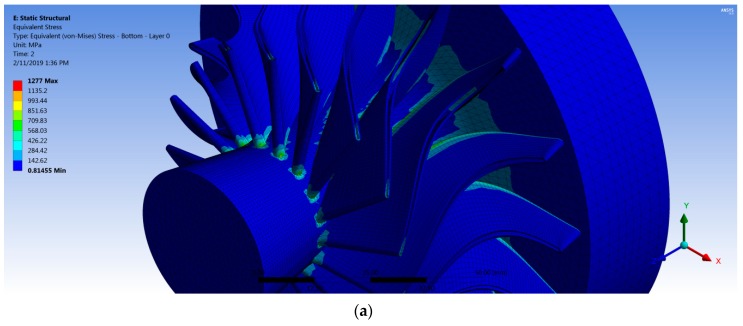

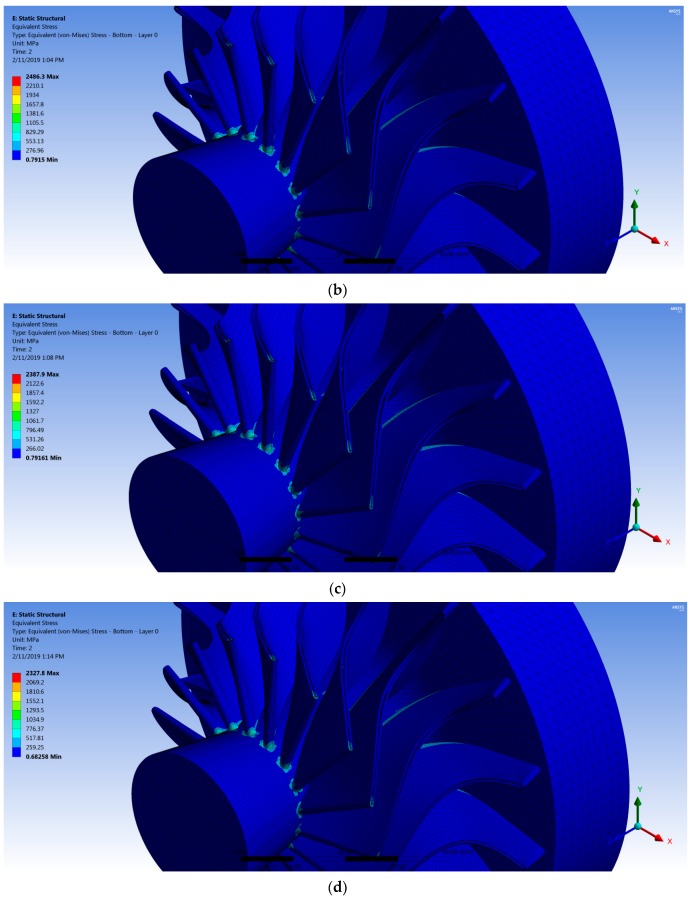

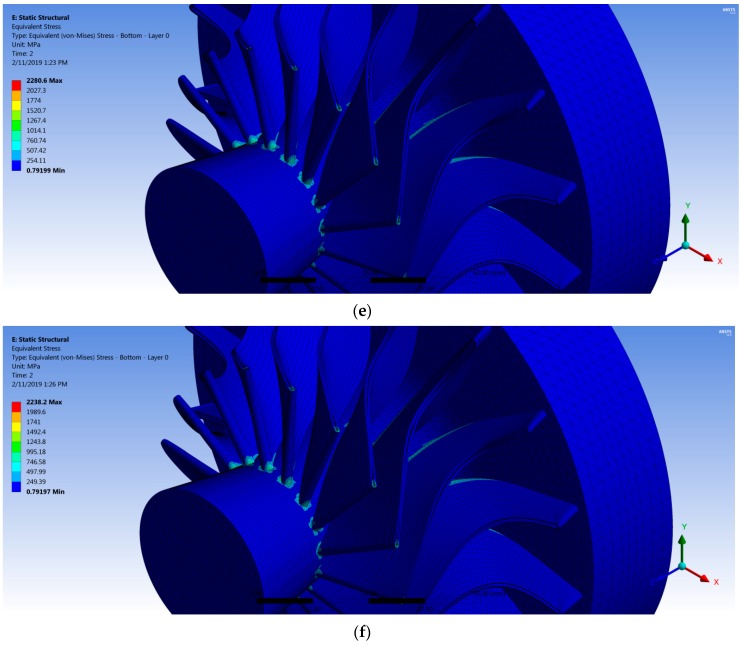
von Mises stress for the turbine with (**a**) Steel rotor blades and (**b**–**f**) a composite rotor with different fiber orientations: (**b**) 20°; (**c**) 30°; (**d**) 40°; (**e**) 50°; and (**f**) 60°.

After importing the forces from CFX into the mechanical module, the structure was solved, and two main results were obtained: blade deflection and von Mises stresses on the turbine wheel. Figure 17 shows the stress contours of the turbine wheel at 40,219 rpm for two cases with different fiber orientation. The maximum stress value was found to be located at the connection between the blade root section and the wheel hub. Thereby, the stress concentration level at the joint can be avoided by increasing the fillet radius. Table 4 shows the maximum stress values for all cases with different fiber orientation.

**Figure 18:**
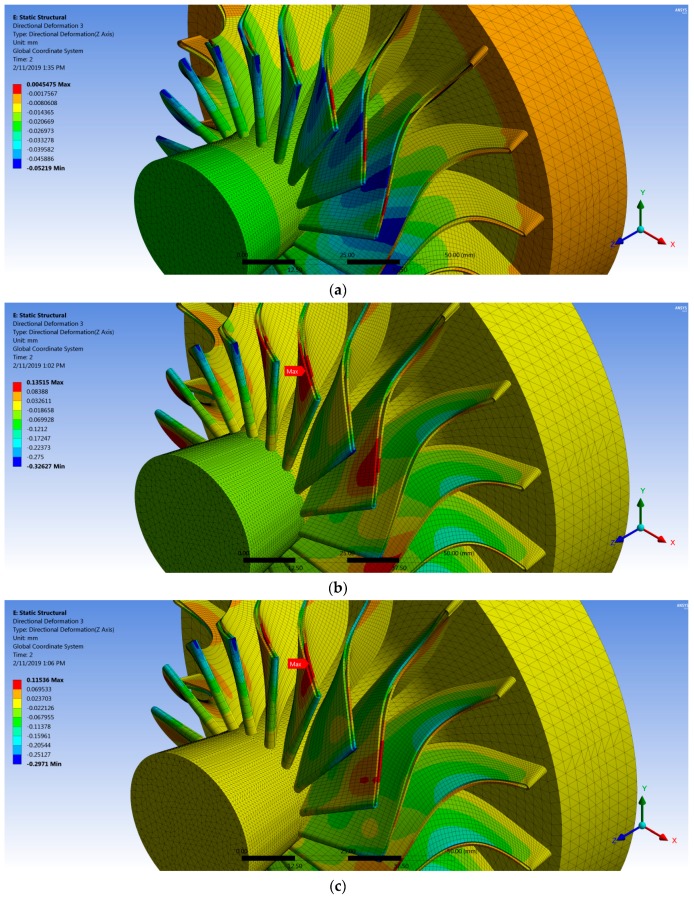

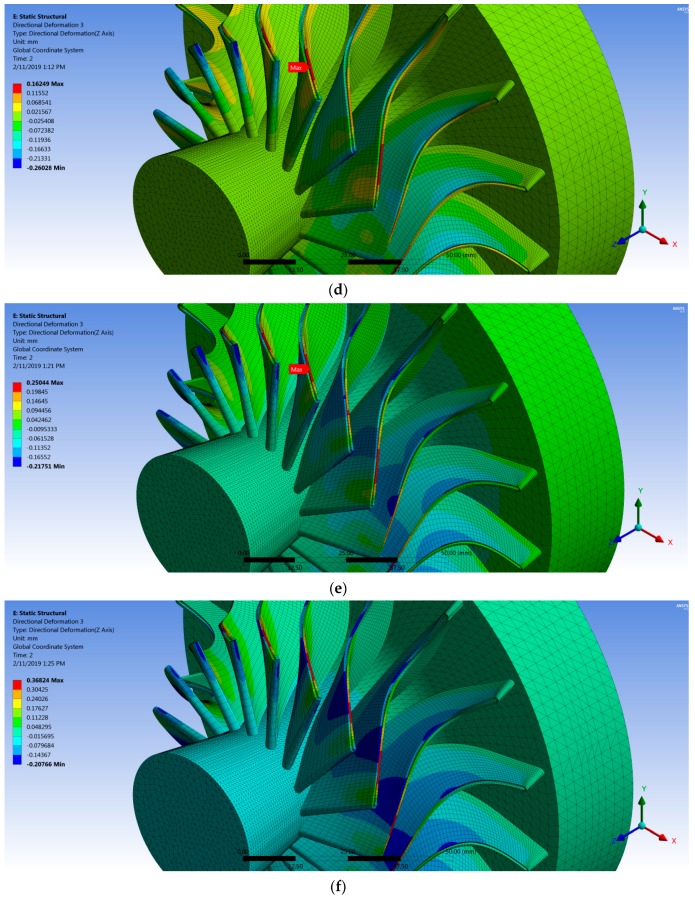
Directional deformation (*z* direction) for (**a**) steel rotor blades and (**b**–**f**) composite rotor with different fiber orientations: (**b**) 20°; (**c**) 30°; (**d**) 40°; (**e**) 50°; and (**f**) 60°.

**Figure 19:**
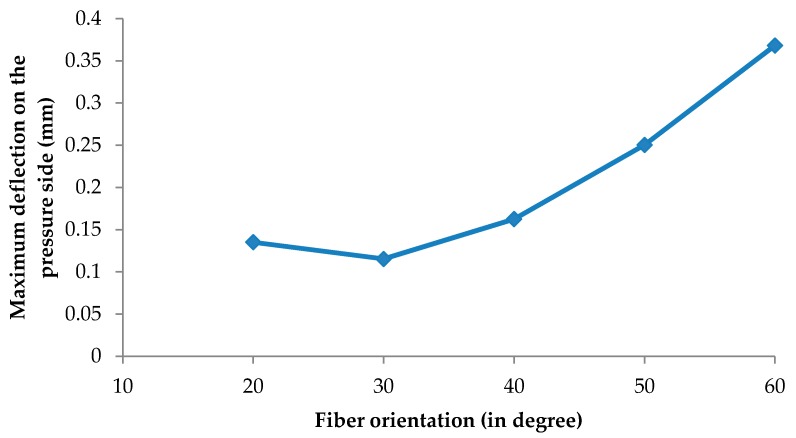
Effect of different fiber orientation on maximum deflection of the pressure side blade.

Figure 18 shows the deformation distribution of the rotor blades for all cases where the maximum value occurred at the shroud of the pressure side near the most curved section due to the high pressure on this side, in addition to the force created by change of the flow momentum. It is worth noticing that the fiber orientation had a significant effect on the deflection of the composite rotor blades, as shown in Table 4, where the deflection was decreased with increasing fiber orientation angle to reach the minimum value of 0.1153 mm (30° fiber orientation), and then the deflection was increased gradually to reach a maximum value of 0.3682 mm (60° fiber orientation) as shown in Figure 19. It can be concluded that the optimum fiber orientation angle (30°) was consistent with the barb angle in the tip leading of the feather (23 ± 7.5°) as aforementioned, consequently the proposed fiber orientation as inspired from bird feathers could contribute positively to make the composite turbine blade more robust.

### 3.3. Blade Weight

Radial turboexpanders are used widely due to their higher specific work. This claim belongs to that the specific work as suggested by Euler work equation for turbomachinery is function of $\frac{1}{2}$($U_{2}{}^{2}-U_{3}{}^{2})$ as shown in Equation (2) [19]:

(2) $$W_{Eu}=\;\frac{1}{2}[(U_{2}{}^{2}-U_{3}{}^{2})-\;(w_{2}{}^{2}-w_{3}{}^{2})\;+\;(c_{2}{}^{2}-c_{3}{}^{2})]$$

where subscripts 2 and 3 represent rotor inlet and outlet, respectively. *U* is the blade speed, *c* is the absolute flow velocity and *w* is the relative velocity between the blade speed and absolute flow velocity.

This term has a positive contribution in the specific work due to higher tip speed in radial turboexpanders. However, higher turbine tip speed increases the centrifugal stresses on the rotor blades. In order to increase the turbine rotational speed without increasing the centrifugal stresses, composite material can be introduced to achieve that. Table 5 shows the significant reduction (80%) in the weight of composite rotor blades compare to that of stainless steel rotor blades which can contribute to increase the extracted specific work by the radial turboexpander. 

## 4. Conclusions

The fluid–structure interaction of the proposed bird-mimetic composite turbine blade was evaluated using CFD and FEA simulations by means of accurately predict the aerodynamic loads and static structural response of the blade. Consequently, the CFD model was validated against the experimental results from a test rig using air. The boundary conditions of the turboexpander switched from air to an organic fluid (R600a) as a result of implementing the turboexpander in an ORC system. Subsequently, a steady-state CFD simulation was performed in order to calculate the aerodynamic and thermal loads that required running FEA simulations to check the feasibility of producing the composite turboexpanders in low-temperature ORCs. Moreover, the structural modeling of the rotor blades was performed to investigate the effect of fiber orientation on their rotor blade deflection due to FSI. The proposed fiber orientation angle inspired from nature has a significant effect to decrease the blade deformation. The main obtained results are summarized as follows:

- The fiber orientation angle has a significant effect on the deformation of the rotor blades and the minimum deflection value was observed with 30° fiber orientation which is consistent with the barb angle at the tip leading of the flight feather. A little change in the fiber orientation has a greater effect on the deformation; the deformation was increased by 219.3% by using 60° fiber orientation compared with the deflection observed at 30° fiber orientation.
- The composite rotor blades weighed 0.0237 kg instead of 0.1234 kg, which means a weight reduction of 80%, and was proven to be structurally robust. This weight reduction can contribute to increasing the turbine rotational speed, which will increase the specific work without increasing the centrifugal stresses on the turbine.

The findings of the current work are in three areas: (i) the orientation of the fiber significantly influences blade deformation; (ii) the use of a composite material leads to a weight reduction of the turbine rotor blades up to 80% vs. stainless steel; and (iii) the bioinspired design of the composite blades of the ORC turbines was found to be the optimum design for the fiber angles as inspired by the bird barb angles. Future work will focus on the experimental verification of implementing these biomimetic blades for ORC turbines. In addition, a numerical investigation of two-way FSI for biomimetic blades will be conducted to determine the efficiency variations with fiber orientations.

## Figures and Tables

**Table 1:** Boundary conditions of two cases for the turboexpander.

Cases	Inlet Turbine Pressure (kPa)	Inlet Turbine Temperature (K)	Outlet Turbine Pressure (kPa)	Rotational Turbine Speed (RPM)
Air	413.6	477.6	72.4	71700
R600a	1872	370	395	40219

**Table 2:** Properties of materials used in modeling the turbine.

Material	Density (kg/m^3^)	E_x_ (MPa)	E_y_ = E_z_ (MPa)	G_yz_ (MPa)	G_xy_ = G_xz_ (MPa)	X_t_ (MPa)	Y_t_ = Z_t_ (MPa)	ν_xy_ = ν_xz_	ν_yz_
Epoxy carbon UD prepreg	1490	121000	8600	3100	4700	2231	29	0.27	0.4
Stainless steel	7750	193000	193000	73664	73664	207	207	0.31	0.31

UD: unidirectional.

**Table 3:** Comparison of total-to-static efficiency and mass flow rate between computational fluid dynamics (CFD) results and experimental data [15].

Case	Mass Flow Tate (kg/s)		Total-to-Static Efficiency (%)	
	CFD	Error (%)	CFD	Error (%)
Jones [15]	0.339	2.7	87.6	1.4

**Table 4:** Maxiumu von Mises stress and tip deflection.

Case	Maximum von Mises Stress (Mpa)	Maximum Deflection on the Presure Side (mm)	Percentage of Deflection from 30° Fiber Orientation (%)
Stainless steel	1277	0.0045	−96.09
20° fiber orientation	2486.3	0.1351	17.17
30° fiber orientation	2387.9	0.1153	-
40° fiber orientation	2327.8	0.1625	40.93
50° fiber orientation	2280.6	0.2504	117.1
60° fiber orientation	2238.2	0.3682	219.3

**Table 5:** Rotor blades weight.

Case	Weight (kg)	Percentage in Reduction of Blades Weight Using Composite Material (%)
Stainless teel rotor blades	0.1234	-
Composite rotor blades	0.0237	80.0
